# Optical coherence tomography in patients with major depressive disorder

**DOI:** 10.1186/s12888-025-06775-7

**Published:** 2025-04-09

**Authors:** Evelyn B.N. Friedel, Malina Beringer, Dominique Endres, Kimon Runge, Simon Maier, Sebastian Küchlin, Jürgen Kornmeier, Katharina Domschke, Sven P. Heinrich, Ludger Tebartz van Elst, Kathrin Nickel

**Affiliations:** 1 Department of Psychiatry and Psychotherapy, Medical Center – University of Freiburg, Faculty of Medicine, University of Freiburg, Freiburg, Germany; 2 Eye Center, Medical Center – University of Freiburg, Faculty of Medicine, University of Freiburg, Freiburg, Germany; 3 Faculty of Biology, University of Freiburg, Freiburg, Germany; 4 Institute for Frontier Areas of Psychology and Mental Health, Freiburg, Germany; 5 German Center for Mental Health (DZPG), Partner Site Berlin, Berlin, Germany

**Keywords:** Optical coherence tomography, OCT, Major depressive disorder, Depression

## Abstract

**Background:**

Optical coherence tomography (OCT) has emerged as a significant non-invasive imaging technique in psychiatric research, allowing for detailed structural assessment of the retinal layers. While OCT has been promising for monitoring neurodegeneration in Alzheimer’s and Parkinson’s diseases, results in major depressive disorder (MDD) remain inconsistent. Building on findings of attenuated electroretinogram (ERG) responses in MDD, indicating functional retinal alterations, this study investigated whether structural retinal changes, such as thinning of retinal layers, also occur in patients with MDD and whether these changes are associated with the severity of depressive symptoms.

**Methods:**

OCT examinations were conducted on 31 MDD patients and 60 healthy controls (HC). The thickness and volumes of macular retinal layers were analyzed and correlated with depressive symptoms.

**Results:**

The thickness of the outer nuclear layer (ONL) was significantly reduced in MDD patients (*p* = 0.003) and negatively correlated with depressive symptoms (BDI-II scores; *p* = 0.001). The thickness and volume of the ganglion cell and inner plexiform layer were further inversely associated with depressive symptoms in MDD patients (BDI-II and MADRS scores; *p <* 0.02).

**Conclusions:**

ONL thinning in MDD suggests possible neurodegenerative processes that may disturb signal transmission downstream, as indicated by altered ERG responses in MDD. Further research is essential to determine the reversibility of these structural changes following remission. Structural retinal analysis with OCT offers the potential to complement functional ERG examinations and represents a promising approach for developing diagnostic and therapeutic monitoring tools for MDD.

**Supplementary Information:**

The online version contains supplementary material available at 10.1186/s12888-025-06775-7.

## Background

Optical coherence tomography (OCT) is a non-invasive imaging method enabling high-resolution cross-sectional visualization of retinal structures [1, 2]. OCT is a routine ophthalmological examination tool for diagnosing and monitoring retinal diseases [3, 4]. As the retina shares an embryological origin with the central nervous system and is considered an extension of the brain [5], it has increasingly gained significance in the study of neurological disorders such as multiple sclerosis [6] or neurodegenerative conditions like Parkinson’s [7–11] and Alzheimer’s disease [12–14]. Therefore, OCT is proposed as a tool to detect markers for neurodegeneration and neuroinflammation [15], both of which are linked to psychiatric diseases like major depressive disorder (MDD) [16–18] or schizophrenia [19, 20].

In an earlier large study, 50 patients with recurrent MDD, 50 with a first episode of MDD, and 50 healthy controls (HC) underwent OCT. Results showed significant reductions in the volumes of the ganglion cell (GCL) and inner plexiform layer (IPL), as well as trend-level thinning in the thickness of the global, temporal and superior retinal nerve fiber layer (RNFL) in patients with recurrent depression compared to those with a first episode of MDD and to HC [21]. In line, a thinner GCL and RNFL were reported to be linked to a higher prevalence of depression [22]. GCL and IPL volume reductions were associated with depression disease severity scores [21] and the duration of the most recent depressive episode [23].

The results suggest that OCT could be a promising tool for detecting and monitoring neurodegeneration in MDD [24, 25]. Concordantly, a thinner peripapillary RNFL (pRNFL) and macular thickness were reported in MDD [26].

In contrast, Sönmez [27] detected no reduction in the overall pRNFL thickness in 30 patients with MDD compared to 30 HC and suggested that unipolar depression has a different underlying pathophysiology than neurodegenerative disorders. Likewise, no differences in the retinal layers were detected in a recent OCT study, assessing 28 patients with MDD and 20 HC. Only a significant difference in total retinal volume between the right and left eye was found in MDD patients and not HC [28].

The significance of potential structural retinal alterations in depression for therapy monitoring or elucidating underlying etiological factors has not yet been fully clarified.

Besides structural retinal changes, previous electrophysiological examinations using flash electroretinogram (fERG) have already demonstrated altered functional integrity of retinal signals in schizophrenia [29–31] and MDD [32, 33]. Moreover, reduced pattern electroretinogram (PERG) contrast gain [34], which normalized following remission [35], and PERG ratio [36] have been observed in MDD and proposed as possible objective marker candidates. Whether structural retinal assessments via OCT, which could potentially complement functional evaluations, may also serve as promising tool for identifying biomarkers or correlates of depressive states was subject of the present investigation.

### Aims of the study

Building on our previous ERG studies demonstrating functional retinal changes in terms of reduced retinal PERG contrast gain, declined PERG ratios and attenuated fERG a-wave amplitudes in patients with depression [33, 34, 36], this study aims to investigate possible structural alterations in retinal layers in MDD and explore their association with depression severity.

## Methods

### Participants

The study was conducted in accordance with the Declaration of Helsinki, following approval from the Ethics Committee of the University of Freiburg (Approval ID: 114/18). Written informed consent was obtained from all participants.

A total of 44 patients with a severe depressive episode and 70 HC were recruited for the present study.

An overlapping cohort was examined using pattern and flash electroretinogram (PERG/fERG), alongside analysis of contrast sensitivity, with findings reported in another publication [33].

Patients were diagnosed with a severe depressive episode according to the criteria of the International Statistical Classification of Diseases, 10th Revision (ICD-10), specifically meeting the criteria for either a severe depressive episode (ICD-10: F32.2) or a recurrent depressive disorder, with a current severe episode without psychotic symptoms (ICD-10: F33.2). The diagnoses were clinically confirmed by an experienced senior specialist in psychiatry and psychotherapy at the Department of Psychiatry and Psychotherapy of the University Medical Center Freiburg. Moreover, the Montgomery-Åsberg Depression Rating Scale (MADRS) [37] was collected. HC were recruited through advertisements and matched to the MDD patients according to age and sex. All study participants completed the following questionnaires: Beck Depression Inventory (BDI-II) [38, 39], Autism Spectrum Quotient (AQ) [40], Empathy Quotient (EQ) [41], Wender Utah Rating Scale (WURS-k) [42], the Structured Clinical Interview for DSM-IV (SCID-I and II) [43], and the Symptom Checklist (SCL-90-R) [44].

The following exclusion criteria were defined: Presence of psychotic symptoms, substance abuse, bipolar disorder, or any psychiatric disorder in the HC group. Antidepressants that were not excluded encompass classes of serotonin–norepinephrine reuptake inhibitors (SNRI) like venlafaxine, selective serotonin reuptake inhibitors (SSRI), such as sertraline as well as noradrenergic and specific serotonergic antidepressants (NaSSAs) like mirtazapine. Additionally, somatic diseases such as diabetes mellitus or arterial hypertension, and the presence of ophthalmological diseases (except for myopia not exceeding − 6 dpt or hyperopia not exceeding + 6 dpt) were exclusion criteria. Only participants with a decimal visual acuity of ≥ 0.8 (logMAR ≤ 0.1) were included.

### Optical coherence tomography

**Fig. 1:**
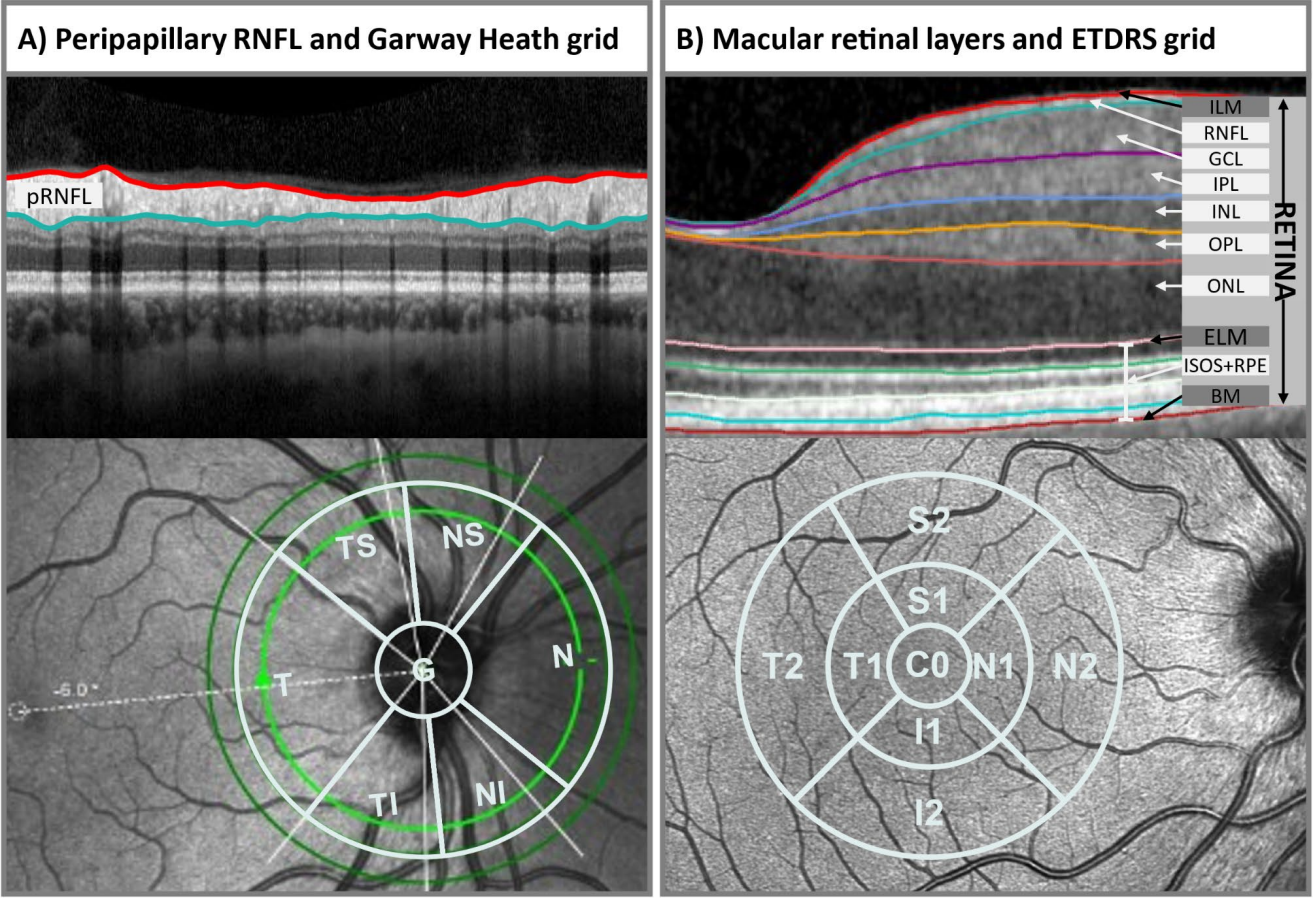
Schematic overview of both OCT scans. **A**: Three circular scans around the optic nerve head for the evaluation of the peripapillary retinal nerve fiber layer (pRNFL) thickness in µm (circle diameters: 3.5, 4.1, and 4.7 mm). The 6 sector Garway-Heath (GH) grid was applied to export thickness data of all sectors including the global thickness (G) as average across all sectors. **B**: Fovea centered macular scan and automated segmentation of the retinal layers. The global volume (mm³) and the thickness data (µm) from the individual 9 sectors of the grid according to the “Early Treatment Diabetic Retinopathy Study” (ETDRS) were exported. Abbreviations: BM = Bruch’s membrane; ELM = external limiting membrane; ETDRS = Early Treatment Diabetic Retinopathy Study; G = global; GCL = ganglion cell layer; I = inferior; ILM = internal limiting membrane; INL = inner nuclear layer; IPL = inner plexiform layer; N = nasal; ONL = outer nuclear layer; OPL = outer plexiform layer; ISOS + RPE = complex of the inner and outer segments of the photoreceptors and the retinal pigment epithelium; pRNFL = peripapillary retinal nerve fiber layer; RNFL = retinal nerve fiber layer; S = superior; T = temporal; 1 = para-foveal sectors; 2 = peri-foveal sectors

The spectral-domain OCT (SD-OCT) measurements were conducted using the Spectralis^®^ OCT (Heidelberg Engineering) with the Glaucoma Module Premium Edition^®^ (version 6.9.4.0). The module includes an Anatomical Positioning System (APS) with automated alignment of predefined scan patterns on the individual anatomical maps of the participants’ eyes. As shown in Fig. 1, two scan patterns were obtained: (1) circular scans around the optic nerve head (ONH) (768 A-scans and 3 circular B-scans: 3.5, 4.1, and 4.7 mm diameter centered on Bruch’s membrane opening) for the evaluation of the peripapillary retinal nerve fiber layer (pRNFL), and (2) a fovea centered macular scan (768 A-scans and 61 B-scans) for the evaluation of the thicknesses and volumes of the different retinal layers.

The automated layer segmentation of the Eye Explorer^®^ software (version 1.10.2.0) was used and individually checked for proper layer detection. In addition, a specialist in ophthalmology (SK) checked all OCT measurements for incidental findings that would require further diagnostic examinations by a specialist.

The HRA/Spectralis^®^ module (version 6.9.5.0) was utilized for data export. Thickness data from the three circle scans of the pRNFL were obtained according to the 6-sector Garway-Heath (GH) grid [45] (Fig. 1) with the “global” thickness (average thickness across sectors) additionally included.

Thickness data of the segmented layers from the macular scan were exported according to the 9-sector grid of the Early Treatment Diabetic Retinopathy Study (ETDRS) [46] (Fig. 1). Additionally, the total volume of the ETDRS grid for each layer was exported. Layers that were considered in the analysis and exported from the macular scan are depicted in Fig. 1 and comprise: the total retina (RETINA) from the internal limiting membrane to the Bruch’s membrane, the retinal nerve fiber layer (RNFL), the ganglion cell layer (GCL), the inner plexiform layer (IPL), the inner nuclear layer (INL), the outer plexiform layer (OPL), the outer nuclear layer (ONL) encompassing the Henle’s Fiber layer (HFL; next to the OPL) and the complex of the inner and outer segments of the photoreceptors (ISOS) and the retinal pigment epithelium (ISOS + RPE).

### Data Preparation and statistical analysis

Data preparation, statistical analysis and graphical representations were performed using “R” in RStudio [47] and the “tidyverse” core packages [48].

Categorical data are presented as the number of observations and their relative frequency (in %). Group comparisons on categorical data between HC and MDD were conducted using a permutation-based chi-squared test (10,000 replicates) with the “infer” package [49].

Continuous data are summarized as mean and standard deviation (SD) with the additional bootstrapped (10,000 replicates) 95% confidence interval, using the “DescTools” package [50].

Decimal visual acuity was assessed with the Freiburg Acuity and Contrast Test [51] and recalculated as the logarithm of the minimum angle of resolution (logMAR = − log_10_(decimal acuity)). For the macular scan, the average (mean) thickness across the 9 sector tiles of the ETDRS grid was calculated for each layer. The Shapiro-Wilk Test was used to test the assumption of normal distribution in all OCT data subsets using the “rstatix” package [52].

Due to the non-normal distribution in some data sets, we used non-parametric permutation tests (10,000 replicates) to compute *p*-values based on the differences in means, utilizing the “infer” package [49]. According to Meissel and Yoa [53], Cliff’s Delta (δ) was calculated as a non-parametric effect size estimation for the difference between MDD and HC, considering |δ| < 0.15 as negligible, |δ| < 0.33 as small, |δ| < 0.47 as medium and |δ| ≥ 0.47 as large effects. In addition, the relative deviation of the MDD means from the HC means was calculated in %.

To streamline statistical analysis and given that there is no reasonable hypothesis suggesting psychiatric disorders should affect the left and right eyes differently, we intended to average the data from both eyes for each participant. This approach avoids the “eye-or-patient problem” caused by the strong correlation between the data of both eyes of an individual [54]. However, because Schönfeldt et al. [28] previously reported a larger retinal volume in the left eye in MDD, we first compared the global pRNFL thickness of the GH grid, the average thicknesses, and total volumes of all macular layers from the ETDRS grid between the right and left eyes separately for both groups. If no significant differences were found, data from both eyes were subsequently averaged.

A significance level of α = 0.05 was defined to indicate statistical significance. All statistical comparisons were carried out without prior directional assumptions (two-sided tests). No correction for multiple testing was applied for comparisons between data from both eyes. For group comparisons between HC and MDD and correlation analyses, a false discovery rate (FDR) procedure [55] was applied for controlling the significance level according to the number of layers evaluated (RETINA, (p)RNFL, GCL, IPL, INL, OPL, ONL, ISOS + RPE).

Spearman’s *rho* was computed (“correlation” package [56]) to assess associations between OCT data and the severity of depressive symptoms (assessed by BDI-II and MADRS scores). The statistical significance of correlation coefficients was tested using permutation tests (10,000 replicates) (“jmuOutlier” package [57]).

## Results

### Participants

Of the initial 44 patients recruited with MDD, six subsequently withdrew from participation, three were excluded due to incidental OCT findings in both eyes, one was excluded due to uncorrectable low visual acuity in both eyes, and three were excluded because they were on the antidepressant medications bupropion, opipramol, or trimipramine. Among the remaining 31 MDD patients, two had low visual acuity (< 0.8) in one eye. Data of these eyes were not included in the analysis.

In the HC group, 10 of the 70 recruited individuals were excluded. Two withdrew, two had poor visual acuity in both eyes, and six incidental OCT findings in both eyes. Out of the remaining 60 HC subjects, four single eyes were excluded, three due to low visual acuity (< 0.8), one because of an OCT finding.

Table 1 depicts demographic and psychometric data of all study participants. Thirty-one patients with MDD (60 eyes; 22 female subjects) and 60 HC (116 eyes; 44 female subjects) matched according to sex and age were included in the final analysis.

Twenty-two (71%) MDD patients took antidepressant medication. One (3%) patient was on sertraline, six (19%) on venlafaxine and six (19%) on mirtazapine. Five (16%) patients were medicated with a combination of mirtazapine and venlafaxine, four (13%) took mirtazapine and sertraline.

**Table 1:** Demographic and psychometric data. Continuous data are summarized as mean and standard deviation (SD), categorical data as number of count and relative frequency in %. *P*-values were calculated using permutation tests, employing Chi-Squared tests for categorical data and differences in means for continuous data. Significance levels are annotated in brackets

Parameter	MDD (*N* = 31)	HC (*N* = 60)	*p*-value
Age in years	31.61 (11.60)	34.42 (12.00)	0.565 (N.S.)
Male/Female	9 (29%)/22 (71%)	16 (27%)/44 (73%)	0.384 (N.S.)
Visual acuity in logMAR	−0.09 (0.09)	−0.10 (0.10)	0.290 (N.S.)
Smoking yes/no	4 (13%)/27 (87%)	6 (10%)/54 (90%)	1.000 (N.S.)
ICD-10 Diagnosis: F32.2/F33.2	16 (52%)/15 (48%)	−	−
Antidepressant medication yes/no	22 (71%)/9 (29%)	−	−
Medication duration in days	7.50 (4.47)	−	−
Venlafaxine	6 (19%)	−	−
Sertraline	1 (3%)	−	−
Mirtazapine	6 (19%)	−	−
Mirtazapine, Venlafaxine	5 (16%)	−	−
Mirtazapine, Sertraline	4 (13%)	−	−
MADRS	37.10 (3.18)	−	−
BDI-II	29.43 (10.49)^+^	2.92 (3.20)^+^	< 0.001 (*)
AQ	18.50 (5.16)^+^	21.27 (3.80)^+^	< 0.001 (*)
EQ	42.50 (7.72)^+^	45.43 (4.84)^++++^	0.160 (N.S.)
WURS-k	15.45 (14.64)^++^	9.31 (7.62)^++^	0.225 (N.S.)

Abbreviations: AQ = Autism Spectrum Quotient; BDI-II = Beck Depression Inventory II; EQ = Empathy Quotient; F32.2 = severe depressive episode without psychotic symptoms (ICD-10); F33.2 = recurrent depressive disorder, current episode severe without psychotic symptoms (ICD-10); HC = healthy controls; ICD-10 = International Statistical Classification of Diseases and Related Health Problems version 10; MADRS = Montgomery-Åsberg Depression Rating Scale; MDD = patients with major depressive disorder; N = number of participants; N.S. = not significant; WURS-k = Wender Utah Rating Scale. ^+^ = 1 missing data set; * = statistically significant difference

### pRNFL thickness from the circle scans around the optic nerve head

Comparing the global pRNFL thickness between the right and left eyes for both groups revealed a small lateralization in the MDD group. Specifically, in the circle scans adjacent to the papilla, the right eye showed a slightly larger pRNFL thickness compared to the left eye (3.5 mm diameter: R vs. L + 1.20%; *p* = 0.004; 4.1 mm diameter: R vs. L: +1.14%; *p* = 0.022). No eye difference in the pRNFL of the MDD patients was observed for the largest circle scan (4.7 mm diameter). Similarly, no differences between the right and left eye data were found for the HC group. (Supplementary Table 1).

Comparing the global pRNFL thickness data between MDD and HC considering all three circle scans and both eyes separately revealed no significant differences between groups (Supplementary Table 1).

### Macular retinal layers from the fovea centered scan

No differences between the right and left eyes in the total volume or in the average thickness data of the retinal layers were found, neither in the MDD, nor in the HC group. Therefore, the participants’ individual averages of the left and right eye data were used for further analysis.

**Fig. 2:**
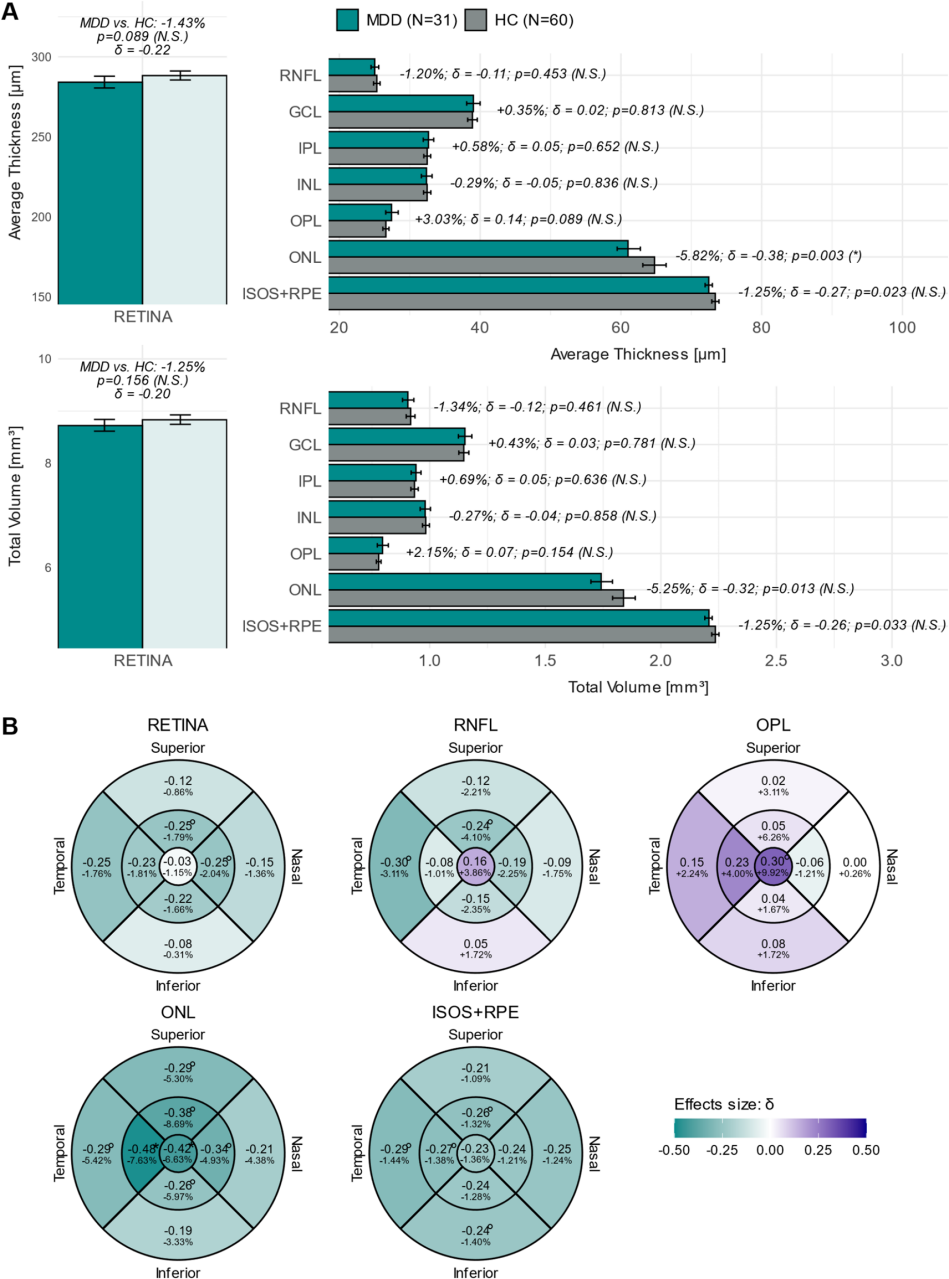
**A**: Means and bootstrapped 95% CIs (error bars) of the average thicknesses (µm) and total volumes (mm³) of all retinal layers for both groups. Relative deviations of the MDDs’ from the HCs’ means in % (MDD vs. HC), Cliff’s delta (δ) as effect size (MDD − HC) and *p*-values from permutation test are annotated. FDR adjusted significance levels according to Benjamini and Hochberg [55] in brackets. **B**: Subset of the ETDRS sector analysis for the thicknesses of the total RETINA, RNFL, OPL, ONL and ISOS + RPE complex. Sector tiles are color-coded based on Cliff’s delta (δ). The relative deviations of the MDD’s from the HC’s means in % are annotated. Superscripted circles (°) indicate *p*-values < 0.05, superscripted stars (*) significant differences between MDD and HC with FDR adjustment included. Abbreviations: ETDRS = Early Treatment Diabetic Retinopathy Study; FDR = false discovery rate; GCL = ganglion cell layer; HC = healthy controls; INL = inner nuclear layer; IPL = inner plexiform layer; MDD = patients with major depressive disorder; N = number of participants; N.S. = not significant; ONL = outer nuclear layer; OPL = outer plexiform layer; ISOS + RPE = complex of the inner and outer segments of the photoreceptors and the retinal pigment epithelium; RNFL = retinal nerve fiber layer; * = statistically significant difference; ° = *p*-value < 0.05

**Fig. 3:**
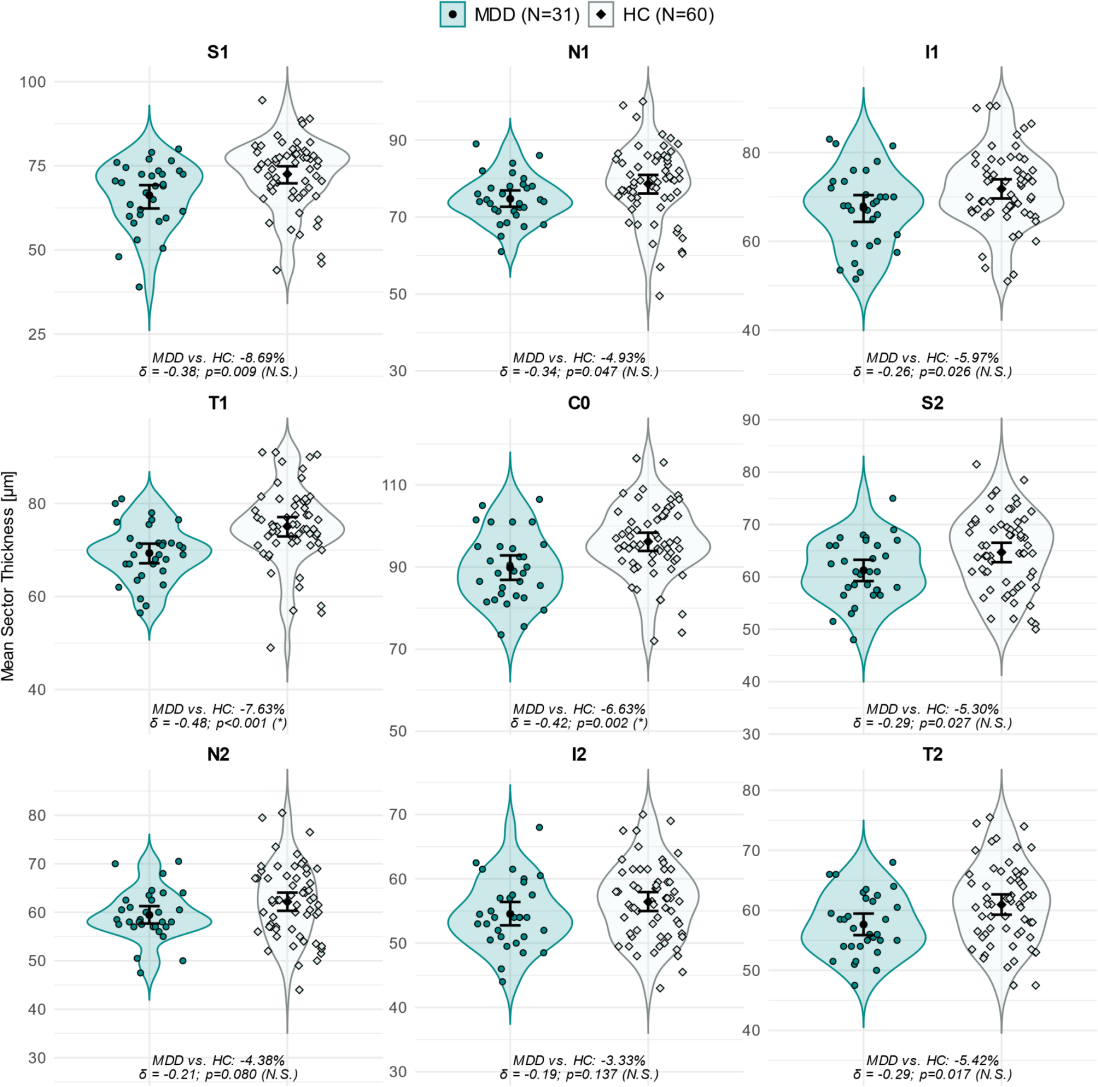
Individual ONL thickness (µm) data from the 9 sectors of the EDTRS grid (compare Fig. 1) for both groups. The means and bootstrapped 95% CIs (error bars) are depicted. Relative deviations of the MDDs’ from the HCs’ means in % (MDD vs. HC), Cliff’s delta (δ) as effect size (MDD − HC) and *p*-values from permutation test are annotated. FDR adjusted significance levels according to Benjamini and Hochberg [55] in brackets. Abbreviations: C0 = foveal sector; ETDRS = Early Treatment Diabetic Retinopathy Study; FDR = false discovery rate; HC = healthy controls; MDD = patients with major depressive disorder; I1/I2 = inferior para-/perifoveal sectors; N = number of participants; N1/N2 = nasal para-/perifoveal sectors; N.S. = not significant; ONL = outer nuclear layer; S1/S2 = superior para-/perifoveal sectors; T1/T2 = temporal para-/perifoveal sectors; * = statistically significant difference

After FDR adjustment a significant reduction in the average ONL thickness in MDD compared to HC (MDD vs. HC: −5.82%, *p* = 0.003) was evident (Fig. 2A, Supplementary Table 2). As evident from the analysis on the individual nine sectors of the ETDRS grid, the ONL reduction was significant and most prominent in the fovea (C0: MDD vs. HC: −6.63%, *p* = 0.002) and in the para-foveal temporal (T1: MDD vs. HC: −7.63%, *p* < 0.001) sectors (Figs. 2B and 3, Supplementary Table 2).

The reduction in the global ONL volume (MDD vs. HC: −5.25%, *p* = 0.013 (N.S.)) and the thickness reduction in the superior, nasal and inferior para- (sectors S1, N1, I1) as well as in the superior and temporal peri-foveal sectors (S2 and T2) must be considered non-significant after FDR adjustment (Figs. 2A-B and 3, Supplementary Table 2).

Similarly, the reduction in the ISOS + RPE complex in MDD compared to HC did not withstand correction for multiple testing (global volume: MDD vs. HC: −1.25%, *p* = 0.033 (N.S.); average thickness: MDD vs. HC: −1.25%, *p* = 0.023 (N.S.); and the sectors: S1, I2, T1, T2) (Fig. 2A-B; Supplementary Table 2).

Likewise, the S1 and N1 sectors of the RETINA, and the S1 and T2 sectors of the RNLF were observed to be thinner in individuals with MDD compared to HCs (*p*-values < 0.05), but these differences must be regarded non-significant after FDR adjustment. The same applies to the foveal C0 sector of the OPL, which showed increased thickness in MDD compared to HC, but this finding also had to be considered non-significant after FDR correction (Fig. 2A-B; Supplementary Table 2).

### Correlation analysis between macular retinal layers and the severity of depressive symptoms

Significant negative correlations between the ONL thickness and volume and the participant’s severity of depressive symptoms according to the BDI-II scores were observed (Table 2).

We further found the average RETINA thickness, the GCL and IPL thicknesses and volumes to be inversely associated with the patient’s depression severity assessed with the MADRS or BDI-II (Table 2). In the MDD group, we additionally found a negative correlation between the average RNFL thickness and the BDI-II scores. The association between the average RNFL thickness and the patients’ MADRS scores must be considered non-significant after FDR adjustment (Table 2).

**Table 2:** Subset of the results from the correlation analysis comparing the average thicknesses (Thick. [µm]) and total volumes (Vol. [mm³]) of the macular retinal layers from the EDTRS grid and the depressive symptoms of all participants assessed with the BDI-II and for MDD patients separately (BDI-II and MADRS scores). Spearman’s *Rho* was calculated as correlation coefficient. *P*-values were calculated using permutation tests. Significance levels in brackets were FDR adjusted according to benjamini and Hochberg [55]. A subset of results is depicted where some layers did show *p*-values close to 0.05

Subjects	Assessment	Layer	Measure	*N*	*Rho*	*p*-value
All participants	BDI-II	ONL	Thick. [µm]	89	-0.33	0.001 (*)
			Vol. [mm³]	89	-0.30	0.005 (*)
Patients with MDD	BDI-II	RETINA	Thick. [µm]	30	-0.42	0.021 (*)
			Vol. [mm³]	30	-0.40	0.029 (N.S.)
		RNFL	Thick. [µm]	30	-0.52	0.004 (*)
			Vol. [mm³]	30	-0.42	0.024 (N.S.)
		GCL	Thick. [µm]	30	-0.44	0.019 (*)
			Vol. [mm³]	30	-0.48	0.007 (*)
		IPL	Thick. [µm]	30	-0.50	0.005 (*)
			Vol. [mm³]	30	-0.50	0.006 (*)
	MADRS	RETINA	Thick. [µm]	31	-0.46	0.010 (*)
			Vol. [mm³]	31	-0.40	0.025 (N.S.)
		RNFL	Thick. [µm]	31	-0.39	0.032 (N.S.)
			Vol. [mm³]	31	-0.34	0.058 (N.S.)
		GCL	Thick. [µm]	31	-0.51	0.003 (*)
			Vol. [mm³]	31	-0.51	0.005 (*)
		IPL	Thick. [µm]	31	-0.52	0.003 (*)
			Vol. [mm³]	31	-0.46	0.012 (*)

Abbreviations: BDI-II = Beck Depression Inventory II; ETDRS = Early Treatment Diabetic Retinopathy Study; FDR = False discovery rate; GCL = ganglion cell layer; IPL = inner plexiform layer; MADRS = Montgomery-Åsberg Depression Rating Scale; N.S. = not significant; N = number of participants; ONL = outer nuclear layer; RNFL = retinal nerve fiber layer; Thick. [µm] = average thickness in µm; Vol. [mm³] = total volume in mm³

## Discussion

The pRNFL and the thicknesses and volumes of the different macular layers were compared between 31 patients with MDD and 60 HC. While the pRNFL was intact, we observed a significant reduction in the average ONL thickness in MDD patients compared to HC. The ONL thickness was negatively correlated with the severity of the participants’ depressive symptoms as measured by BDI-II scores. Although the MDD group showed no significant thinning of the RETINA or other macular layers compared to the HC group, the patients’ depressive symptoms (according to MADRS and BDI-II scores) were significantly negatively correlated with total volumes and average thicknesses of the GCL and IPL and the average RETINA thickness. Moreover, the mean RNFL thickness was found to be significantly inversely associated with the patients’ BDI-II scores.

Our findings align with previous reports of inverse associations between the GCL and IPL volumes and depressive symptoms in patients with affective disorders [21].

In addition, Kalenderoglu et al. [21] reported volume loss in the GCL and IPL in MDD, along with a reduction in RNFL thickness. In contrast, Schönfeldt-Lecuona et al. [28] found no differences in the retinal volumes or thicknesses between MDD and HC, which is consistent with the results of Sönmez et al. [27], Yildiz et al. [23] and also our findings, apart from the thickness reduction in the ONL.

Possible reasons for the differing results may include variations in OCT equipment (Spectralis OCT [21, 27, 28] vs. Cirrus HD-OCT [23]) and scanning protocols, or the heterogeneity across the investigated populations regarding the patient age, and the duration and severity of the depressive episodes. Kalenderoglu et al. [21] additionally performed a subgroup analysis comparing patients with recurrent MDD to those with a first episode. They found more pronounced reductions in the GCL and IPL in patients with recurrent depressive episodes compared to those with a first depressive episode and suggested that this may be indicative of cumulative neurodegenerative processes [21, 25]. Since the authors did not evaluate the ONL layer it remains unresolved whether ONL thinning was also apparent in their MDD population. Only Schönfeldt-Lecuona et al. [28] assessed the ONL thickness in MDD but could not find differences between patients and HC. Their MDD patients were about 15 years older compared to our patients and all received antidepressants, which were not further differentiated, potentially contributing to the variations. This indicates that future research should include larger and more homogenous populations regarding the medication status and the severity of depressive symptoms.

The ONL is regarded as a quantitative biomarker for photoreceptor function, with thinning indicative of photoreceptor degeneration and potential progression in dry age-related macular degeneration (AMD) [58]. Chronic inflammation and oxidative stress, which are associated with AMD pathogenesis [59, 60], are also linked to neurodegenerative diseases [61].

Similar ONL reductions have been observed in schizophrenia [31], adults with autism spectrum disorders [62] and Parkinson’s disease [9], suggesting possible common neurodegenerative processes. However, this also indicates that ONL thinning does not appear to represent a disease-specific biomarker.

Despite similar structural retinal changes (ONL thinning) in MDD, schizophrenia, autism spectrum disorders and Parkinson’s disease, the functional retinal signals differed. Twenty-seven (87%) of the patients with MDD and 39 HC (65%) who took part in this study also received fERG and PERG evaluations with the results previously reported [33]. For those participants, we observed a reduction in the fERG a-wave amplitude, an attenuated PERG ratio and diminished contrast sensitivity [33]. Previous studies also reported diminished PERG contrast gain [34] and PERG ratio [36] in MDD, indicating impaired GCL responses, which appeared to be stimulus-specific since the PhNR (another correlate for GCL function) in MDD remained preserved [33]. Conversely, adults with autism spectrum disorders exhibited normal fERG responses [63], while both the a-wave and the PhNR were attenuated in patients with schizophrenia [31].

Thus, the integration of structural (OCT) and functional electrophysiological measurements (fERG, PERG) may have the potential to disentangle the underlying pathophysiologies and etiologies and develop into potential diagnostic or therapeutic monitoring tools (see Supplementary Fig. 2).

Supporting the dopamine hypothesis [64] in MDD, recent findings from Parkinson’s disease research are relevant. Tran et al. [9] observed similar attenuated a-wave amplitudes, ONL thinning, and decreased center-surround contrast suppression in patients with Parkinson’s disease compared to HC, reflecting our findings in MDD (reduced a-wave amplitude, ONL thinning, and decreased contrast sensitivity) [33].

Moreover, OCT-Angiography (OCT-A) could be promising as an adjunct to OCT. Current investigations found reduced vessel density of superficial and deep capillary plexus and a decreased volume of the optic nerve head in MDD compared to HC [65]. In line, Xiao [66] confirmed findings of a declined macular vessel density in the superficial retinal capillary plexus in MDD. This points toward neurovascular impairment with the OCT-A being a valuable complementary tool alongside the traditional OCT to investigate patients with psychiatric disorders [65, 66]. Inflammatory processes, potentially linked to increased susceptibility to MDD [67], may cause microvascular blockage and endothelial inflammation, resulting in decreased retinal blood supply and flow velocity [66, 68].

Since the retina, as an extension of the CNS, provides valuable insights into functional and structural changes in MDD, analysis of the retinal neuronal anatomy and function holds promise for diagnosing and monitoring psychiatric disorders, detecting neurodegeneration markers, and differentiating stages of disease progression [69].

### Limitations

Because information about the first onset of MDD and the duration of the latest depressive episode could not reliably be collected, we were not able to draw conclusions about the relationship between the progression of retinal thinning and the duration of the disease. Future studies should incorporate this aspect in more detail to clarify whether ONL thinning represents an early sign of neuronal degeneration with further progression in other retinal layers, and additionally, should include remitted patients to determine the potential reversibility of retinal layer thinning.

The observation that the ONL is significantly reduced in MDD, while the ISOS + RPE shows only trend-level reductions (non-significant after correction), may result either from selective neurodegenerative processes, metabolic dysfunction, or neurotransmitter imbalances primarily affecting photoreceptor cell bodies, or alternatively from the continuous renewal of photoreceptor outer segments by the RPE [70], which compensates and helps preserve the ISOS + RPE layer.

Since 71% of the patients with MDD were taking antidepressant medication, but the sample sizes for the subgroups of medicated (*N* = 22) and unmedicated (*N* = 9) patients with MDD were insufficient for robust inferential comparisons, we cannot exclude the effects of antidepressant medication. Nevertheless, we compared these subgroups descriptively and observed a reduction in the average thickness of the ONL in both medicated (vs. HC: −5.06%) and unmedicated (vs. HC: −7.67%) patients with MDD compared to HC (Supplementary Fig. 1A).

Likewise, a descriptive subgroup analysis was conducted to compare MDD patients with a first severe depressive episode (F32.2; *N* = 16) and those with a recurrent severe depressive episode (F33.2; *N* = 15) separately to HC (Supplementary Fig. 1B). Both subgroups exhibited a similar degree of ONL thinning, regardless of whether the current diagnosis was a first episode (vs. HC: −6.61%) or a recurrent episode (vs. HC: −4.97%).

For future studies, it is important to examine not only a larger cohort of unmedicated MDD patients, but also patients with a standardized medication and medication duration in order to investigate homogeneous subgroups and exclude confounding effects.

## Conclusions

We observed significant ONL thinning in MDD patients compared to HC. ONL thickness was negatively correlated with depressive symptoms according to the BDI-II questionnaire. Further research is needed to elucidate the underlying pathophysiology and establish OCT as a potential diagnostic or therapeutic monitoring tool for MDD. The combination of functional (fERG and PERG) and structural (OCT and OCT-A) ophthalmic examination methods appears particularly promising.

## Electronic supplementary material

Below is the link to the electronic supplementary material.

Supplementary Material 1

## Data Availability

Data collected and/or analyzed in this study can be obtained from the corresponding author and EF upon reasonable request.

## References

[1] Podoleanu AGh. Optical coherence tomography. J Microsc. 2012;247(3):209–19. Available from: 10.1111/j.1365-2818.2012.03619.x 10.1111/j.1365-2818.2012.03619.x PMC3563006 22708800

[2] Aumann S, Donner S, Fischer J, Müller F. Optical Coherence Tomography (OCT): Principle and technical realization. In: Bille JF, editor. High Resolution Imaging in Microscopy and Ophthalmology. Cham: Springer International Publishing 2019; pp. 59–85. Available from: 10.1007/978-3-030-16638-0_3 32091846

[3] Jaffe GJ, Caprioli J. Optical coherence tomography to detect and manage retinal disease and glaucoma. Am J Ophthalmol. 2004;137(1):156–69. 10.1016/s0002-9394(03)00792-x 14700659 10.1016/s0002-9394(03)00792-x

[4] Ţălu SD. Optical Coherence Tomography in the diagnosis and monitoring of retinal diseases. ISRN Biomed Imaging. 2013;2013:1–13. 10.1155/2013/910641

[5] Reichenbach A, Bringmann A, Retina. Neuroanatomy and Physiology. In: Pfaff DW, Volkow ND, Rubenstein JL, editors. Neuroscience in the 21st Century: From Basic to Clinical. Springer International Publishing; 2022;955–1027. Available from: 10.1007/978-3-030-88832-9_22

[6] Filippatou A, Theodorou A, Stefanou MI, Tzanetakos D, Kitsos D, Moschovos C, et al. Optical coherence tomography and angiography in multiple sclerosis: A systematic review and meta-analysis. J Neurol Sci. 2025;470:123422. Available from: 10.1016/j.jns.2025.123422 39954575 10.1016/j.jns.2025.123422

[7] Lee JY, Ahn J, Kim T, Jeon B. Optical coherence tomography in Parkinson’s disease: is the retina a biomarker? J Park Dis. 2014;4(2):197–204. Available from: 10.3233/JPD-130306 10.3233/JPD-130306 24518436

[8] Zhao Y, Zhao J, Gu Y, Chen B, Guo J, Xie J et al. Outer retinal layer thickness changes in white matter hyperintensity and Parkinson’s disease. Front Neurosci. 2021;15:741651. 10.3389/fnins.2021.741651 10.3389/fnins.2021.741651 PMC8477009 34594186

[9] Tran KKN, Lee PY, Finkelstein DI, McKendrick AM, Nguyen BN, Bui BV et al. Altered outer retinal structure, electrophysiology and visual perception in Parkinson’s disease. J Park Dis. 2024;14(1):167–80. Available from: 10.3233/jpd-230293 10.3233/JPD-230293 PMC10836541 38189711

[10] Tebartz van Elst L, Greenlee MW, Foley JM, Lucking CH. Contrast detection, discrimination and adaptation in patients with Parkinson’s disease and multiple system atrophy. Brain J Neurol. 1997;120(Pt 12):2219–28. 10.1093/brain/120.12.2219 10.1093/brain/120.12.2219 9448577

[11] Langheinrich T, Tebartz van Elst L, Lagrèze WA, Bach M, Lücking CH, Greenlee MW. Visual contrast response functions in Parkinson’s disease: evidence from electroretinograms, visually evoked potentials and psychophysics. Clin Neurophysiol. 2000;111(1):66–74. 10.1016/s1388-2457(99)00223-0 10.1016/s1388-2457(99)00223-0 10656512

[12] Lu Y, Li Z, Zhang X, Ming B, Jia J, Wang R, et al. Retinal nerve fiber layer structure abnormalities in early Alzheimer’s disease: evidence in optical coherence tomography. Neurosci Lett. 2010;480(1):69–72. 10.1016/j.neulet.2010.06.006 20609426 10.1016/j.neulet.2010.06.006

[13] Cunha LP, Almeida ALM, Costa-Cunha LVF, Costa CF, Monteiro MLR. The role of optical coherence tomography in Alzheimer’s disease. Int J Retina Vitr. 2019;2(1). 10.1186/s40942-016-0049-4 10.1186/s40942-016-0049-4 PMC5088456 27847642

[14] Chan VTT, Sun Z, Tang S, Chen LJ, Wong A, Tham CC et al. Spectral-Domain OCT measurements in Alzheimer’s disease: A systematic review and meta-analysis. Ophthalmology. 2019;126(4):497–510. 10.1016/j.ophtha.2018.08.009 10.1016/j.ophtha.2018.08.009 PMC6424641 30114417

[15] Vujosevic S, Parra MM, Hartnett ME, O’Toole L, Nuzzi A, Limoli C et al. Optical coherence tomography as retinal imaging biomarker of neuroinflammation/neurodegeneration in systemic disorders in adults and children. Eye. 2023;37(2):203–19. 10.1038/s41433-022-02056-9 10.1038/s41433-022-02056-9 PMC9012155 35428871

[16] Wuwongse S, Chang RCC, Law ACK. The putative neurodegenerative links between depression and Alzheimer’s disease. Prog Neurobiol. 2010;91(4):362–75. 10.1016/j.pneurobio.2010.04.005 10.1016/j.pneurobio.2010.04.005 20441786

[17] Arnone D, McIntosh AM, Ebmeier KP, Munafò MR, Anderson IM. Magnetic resonance imaging studies in unipolar depression: Systematic review and meta-regression analyses. Eur Neuropsychopharmacol. 2012;22(1):1–16. 10.1016/j.euroneuro.2011.05.003 10.1016/j.euroneuro.2011.05.003 21723712

[18] Hurley LL, Tizabi Y, Neuroinflammation. Neurodegeneration, and Depression. Neurotox Res. 2013;23(2):131–44. Available from: 10.1007/s12640-012-9348-1http://link.springer.com/10.1007/s12640-012-9348-1 PMC3751583 22895696

[19] Polho GB, Paula VDJRD. Schizophrenia: neuroinflammation, neurodegeneration or neurodevelopment? A genetic overview. Rev Med. 2017;96(1):39–48. 10.11606/issn.1679-9836.v96i1p39-48

[20] Stone WS, Phillips MR, Yang LH, Kegeles LS, Susser ES, Lieberman JA. Neurodegenerative model of schizophrenia: Growing evidence to support a revisit. Schizophr Res. 2022;243:154–62. 10.1016/j.schres.2022.03.004 10.1016/j.schres.2022.03.004 PMC9189010 35344853

[21] Kalenderoglu A, Çelik M, Sevgi-Karadag A, Egilmez OB. Optic coherence tomography shows inflammation and degeneration in major depressive disorder patients correlated with disease severity. J Affect Disord. 2016;204:159–65. 10.1016/j.jad.2016.06.039 10.1016/j.jad.2016.06.039 27344626

[22] Huang Y, Pan Z, Wang YX, Jonas JB. The impact of depression on individual retinal thickness, the Beijing eye study. Invest Ophthalmol Vis Sci. 2023;64(8):3736–3736.

[23] Yıldız M, Alim S, Batmaz S, Demir S, Songur E, Ortak H et al. Duration of the depressive episode is correlated with ganglion cell inner plexifrom layer and nasal retinal fiber layer thicknesses: Optical coherence tomography findings in major depression. Psychiatry Res Neuroimaging. 2016;251:60–6. 10.1016/j.pscychresns.2016.04.011 10.1016/j.pscychresns.2016.04.011 27124425

[24] Kalenderoglu A, Sevgi-Karadag A, Celik M, Egilmez OB, Han-Almis B, Ozen ME. Can the retinal ganglion cell layer (GCL) volume be a new marker to detect neurodegeneration in bipolar disorder? Compr Psychiatry. 2016;67:66–72. 10.1016/j.comppsych.2016.02.005 10.1016/j.comppsych.2016.02.005 27095337

[25] Clémence-Fau M, Schwan R, Angioi-Duprez K, Laprévote V, Schwitzer T. Retinal structural changes in mood disorders: The optical coherence tomography to better understand physiopathology? Prog Neuropsychopharmacol Biol Psychiatry. 2021;108:110080. 10.1016/j.pnpbp.2020.110080 10.1016/j.pnpbp.2020.110080 32827610

[26] Liu Y, Tong Y, Huang L, Chen J, Yan S, Yang F. Factors related to retinal nerve fiber layer thickness in bipolar disorder patients and major depression patients. BMC Psychiatry. 2021;21(1):301. 10.1186/s12888-021-03270-7 34112131 10.1186/s12888-021-03270-7 PMC8191183

[27] Sönmez İ, Köşger F, Aykan Ü. Retinal nerve fiber layer thickness measurement by Spectral-Domain optical coherence tomography in patients with major depressive disorder. Noro Psikiyatri Arsivi. 2017;54(1):62–6. 10.5152/npa.2015.10115 28566961 10.5152/npa.2015.10115 PMC5439474

[28] Schönfeldt-Lecuona C, Schmidt A, Kregel T, Kassubek J, Dreyhaupt J, Freudenmann RW, et al. Retinal changes in patients with major depressive disorder - A controlled optical coherence tomography study. J Affect Disord. 2018;227:665–71. 10.1016/j.jad.2017.11.077 29174740 10.1016/j.jad.2017.11.077

[29] Hébert M, Mérette C, Paccalet T, Émond C, Gagné AM, Sasseville A et al. Light evoked potentials measured by electroretinogram may tap into the neurodevelopmental roots of schizophrenia. Schizophr Res. 2015;162(1–3):294–5. 10.1016/j.schres.2014.12.030 10.1016/j.schres.2014.12.030 25579051

[30] Hébert M, Mérette C, Gagné AM, Paccalet T, Moreau I, Lavoie J, et al. The electroretinogram May differentiate schizophrenia from bipolar disorder. Biol Psychiatry. 2020;87(3):263–70. 10.1016/j.biopsych.2019.06.014 31443935 10.1016/j.biopsych.2019.06.014

[31] Friedel EBN, Hahn HT, Maier S, Küchlin S, Reich M, Runge K et al. Structural and functional retinal alterations in patients with paranoid schizophrenia. Transl Psychiatry. 2022;12(1):402. 10.1038/s41398-022-02167-7 10.1038/s41398-022-02167-7 PMC9508100 36151078

[32] Hébert M, Mérette C, Paccalet T, Gagné AM, Maziade M. Electroretinographic anomalies in medicated and drug free patients with major depression: Tagging the developmental roots of major psychiatric disorders. Prog Neuropsychopharmacol Biol Psychiatry. 2017;75:10–5. 10.1016/j.pnpbp.2016.12.002 10.1016/j.pnpbp.2016.12.002 28007463

[33] Friedel EBN, Van Tebartz L, Beringer M, Endres D, Runge K, Maier S et al. Reduced contrast sensitivity, pattern electroretinogram ratio, and diminished a-wave amplitude in patients with major depressive disorder. Eur Arch Psychiatry Clin Neurosci. 2024; Available from: 10.1007/s00406-024-01826-8https://link.springer.com/10.1007/s00406-024-01826-8 PMC12149264 38805071

[34] Bubl E, Kern E, Ebert D, Bach M, van Tebartz L. Seeing gray when feeling blue? Depression can be measured in the eye of the diseased. Biol Psychiatry. 2010;68(2):205–8. 10.1016/j.biopsych.2010.02.009 10.1016/j.biopsych.2010.02.009 20359698

[35] Bubl E, Ebert D, Kern E, van Elst LT, Bach M. Effect of antidepressive therapy on retinal contrast processing in depressive disorder. Br J Psychiatry. 2012;201(2):151–8. Available from: 10.1192/bjp.bp.111.100560http://bjp.rcpsych.org/cgi/doi/10.1192/bjp.bp.111.100560 22700080

[36] Friedel EBN, Tebartz van Elst L, Schmelz C, Ebert D, Maier S, Endres D, et al. Replication of reduced pattern electroretinogram amplitudes in depression with improved recording parameters. Front Med. 2021;8:732222. Available from: https://www.frontiersin.org/articles/10.3389/fmed.2021.732222/full 10.3389/fmed.2021.732222 PMC8585854 34778295

[37] Montgomery SA, Åsberg M. A new depression scale designed to be sensitive to change. Br J Psychiatry J Ment Sci. 1979;134:382–9. 10.1192/bjp.134.4.382 10.1192/bjp.134.4.382 444788

[38] Beck AT, Steer RA, Brown G. Manual for the Beck depression Inventory-II. San Antonia, TX: Psychological Corporation; 1996.

[39] Hautzinger M, Keller F, Kühner C. Beck Depressions-Inventar: BDI II. Revision. Deutsche Bearbeitung von Beck, A. T., Steer, R. A., & Brown, G. K. (1996). Beck Depression Inventory–II (BDI–II). Frankfurt am Main: Harcourt Test Services; 2006. Testmappe.

[40] Baron-Cohen S, Wheelwright S, Skinner R, Martin J, Clubley E. The autism-spectrum quotient (AQ): evidence from asperger syndrome/high-functioning autism, males and females, scientists and mathematicians. J Autism Dev Disord. 2001;31(1):5–17. 10.1023/a:1005653411471 11439754 10.1023/a:1005653411471

[41] Baron-Cohen S, Wheelwright S. The empathy quotient: an investigation of adults with Asperger syndrome or high functioning autism, and normal sex differences. J Autism Dev Disord. 2004;34(2):163–75. Available from: 10.1023/B:JADD.0000022607.19833.00https://link.springer.com/article/10.1023/b:jadd.0000022607.19833.00 15162935

[42] Retz-Junginger P, Retz W, Blocher D, Weijers HG, Trott GE, Wender PH, et al. Wender Utah rating scale (WURS-k) die Deutsche Kurzform Zur retrospektiven erfassung des hyperkinetischen syndroms Bei erwachsenen. Nervenarzt. 2002;73(9):830–8. 10.1007/s00115-001-1215-x 12215873 10.1007/s00115-001-1215-x

[43] Wittchen HU, Zaudig M, Fydrich T. SKID. Strukturiertes Klinisches Interview für DSM-IV. Achse I und II. Handanweisung. 1997.

[44] Derogatis LR, Savitz KL. The SCL-90-R, brief symptom inventory, and matching clinical rating scales. In M.E. Maruish (Ed.) The use of psychological testing for treatment planning and outcomes assessment. 2nd ed. Mahwah, NJ, US: Lawrence Erlbaum Associates; 1999. pp. 679–724.

[45] Garway-Heath DF, Poinoosawmy D, Fitzke FW, Hitchings RA. Mapping the visual field to the optic disc in normal tension glaucoma eyes. Ophthalmology. 2000;107(10):1809–15. 10.1016/s0161-6420(00)00284-0 10.1016/s0161-6420(00)00284-0 11013178

[46] Chew EY. Association of elevated serum lipid levels with retinal hard exudate in diabetic retinopathy: Early Treatment Diabetic Retinopathy Study (ETDRS) Report 22. Arch Ophthalmol. 1996;114(9):1079. 10.1001/archopht.1996.01100140281004 10.1001/archopht.1996.01100140281004 8790092

[47] Posit team. RStudio: Integrated Development Environment for R [Internet]. Boston, MA: Posit Software, PBC. 2023. Available from: http://www.posit.co/

[48] Wickham H, Averick M, Bryan J, Chang W, McGowan LD, François R, et al. Welcome to the tidyverse. J Open Source Softw. 2019;4(43):1686. 10.21105/joss.01686

[49] Couch SP, Bray AP, Ismay C, Chasnovski E, Baumer BS, Çetinkaya-Rundel M. Infer: an R package for tidyverse-friendly statistical inference. J Open Source Softw. 2021;6(65):3661. 10.21105/joss.03661

[50] Signorell A, DescTools. Tools for Descriptive Statistics [Internet]. 2023. Available from: https://CRAN.R-project.org/package=DescTools

[51] Bach M. The Freiburg visual acuity Test - Variability unchanged by post-hoc re-analysis. Graefes Arch Clin Exp Ophthalmol. 2007;245(7):965–71. 10.1007/s00417-006-0474-4 17219125 10.1007/s00417-006-0474-4

[52] Kassambara A, rstatix. Pipe-friendly framework for basic statistical tests [Internet]. 2023. Available from: https://CRAN.R-project.org/package=rstatix

[53] Meissel K, Yao E. Using Cliff’s Delta as a Non-Parametric Effect Size Measure: An Accessible Web App and R Tutorial. 2024. 10.7275/PARE.1977

[54] Holopigian K, Bach M. A primer on common statistical errors in clinical ophthalmology. Doc Ophthalmol. 2010 Dec;121(3):215–22. Available from: 10.1007/s10633-010-9249-7http://link.springer.com/10.1007/s10633-010-9249-7 20972602

[55] Benjamini Y, Hochberg Y. Controlling the False Discovery Rate: A practical and powerful approach to multiple testing. J R Stat Soc Ser B Methodol [Internet]. 1995;57(1):289–300. Available from: http://www.jstor.org/stable/2346101

[56] Makowski D, Ben-Shachar M, Patil I, Lüdecke D. Methods and algorithms for correlation analysis in R. J Open Source Softw. 2020;5(51):2306. 10.21105/joss.02306

[57] Garren ST, jmuOutlier. Permutation Tests for Nonparametric Statistics [Internet]. 2019. Available from: https://CRAN.R-project.org/package=jmuOutlier

[58] Lee S, Kim KT, Kim DY, Chae JB, Seo EJ. Outer nuclear layer recovery as a predictor of visual prognosis in type 1 choroidal neovascularization of neovascular age-related macular degeneration. Sci Rep. 2023;13(1):5045. 10.1038/s41598-023-32184-5 36977729 10.1038/s41598-023-32184-5 PMC10050211

[59] Fleckenstein M, Keenan TDL, Guymer RH, Chakravarthy U, Schmitz-Valckenberg S, Klaver CC, et al. Age-related macular degeneration. Nat Rev Dis Primer. 2021;7(1):31. 10.1038/s41572-021-00265-2 10.1038/s41572-021-00265-2 PMC12878645 33958600

[60] Rosa JGS, Disner GR, Pinto FJ, Lima C, Lopes-Ferreira M. Revisiting retinal degeneration hallmarks: Insights from molecular markers and therapy perspectives. Int J Mol Sci. 2023;24(17):13079. 10.3390/ijms241713079 10.3390/ijms241713079 PMC10488251 37685886

[61] Houldsworth A. Role of oxidative stress in neurodegenerative disorders: a review of reactive oxygen species and prevention by antioxidants. Brain Commun. 2023;6(1):fcad356. Available from: 10.1093/braincomms/fcad356/7504872https://academic.oup.com/braincomms/article/doi/10.1093/braincomms/fcad356 PMC10783645 38214013

[62] Friedel EBN, Tebartz van Elst L, Schäfer M, Maier S, Runge K, Küchlin S, et al. Retinal thinning in adults with autism spectrum disorder. J Autism Dev Disord. 2022;54(3):1143–56. Available from: https://link.springer.com/. 10.1007/s10803-022-05882-8 10.1007/s10803-022-05882-8 PMC10907434 36550331

[63] Friedel EBN, Schäfer M, Endres D, Maier S, Runge K, Bach M et al. Electroretinography in adults with high-functioning autism spectrum disorder. Autism Res. 2022;15(11):2026–37. Available from: 10.1002/aur.2823https://onlinelibrary.wiley.com/doi/10.1002/aur.2823 36217563

[64] Dunlop BW, Nemeroff CB. The role of dopamine in the pathophysiology of depression. Arch Gen Psychiatry. 2007;64(3):327. 10.1001/archpsyc.64.3.327 10.1001/archpsyc.64.3.327 17339521

[65] Wang Y, Li C, Liu L, Yang Y, He X, Li G, et al. Association of retinal neurovascular impairment with disease severity in patients with major depressive disorder: an optical coherence tomography angiography study. Psychol Res Behav Manag. 2024;17:1573–85. 10.2147/PRBM.S443146 10.2147/PRBM.S443146 PMC11015850 38617578

[66] Xiao Q, Li F, Jiang F, Zhang Z, Xu B. The prospects for early detection with optical coherence tomography (OCT) and OCT angiography in major depressive disorder. J Affect Disord. 2024;347:8–14. 10.1016/j.jad.2023.11.031 10.1016/j.jad.2023.11.031 37984693

[67] Beurel E, Toups M, Nemeroff CB. The bidirectional relationship of depression and inflammation. Neuron. 2020;107(2):234–56. 10.1016/j.neuron.2020.06.002 10.1016/j.neuron.2020.06.002 PMC7381373 32553197

[68] Lu Y, Tao R, Huang J, Li L, Liao M, Li X, et al. P2X7 signaling promotes microsphere embolism-triggered microglia activation by maintaining elevation of Fas ligand. J Neuroinflammation. 2012;9:172. 10.1186/1742-2094-9-172 10.1186/1742-2094-9-172 PMC3420259 22789015

[69] Tan A, Schwitzer T, Conart JB, Angioi-Duprez K. Study of retinal structure and function in patients with major depressive disorder, bipolar disorder or schizophrenia: a review of the literature. J Fr Ophtalmol. 2020;43(5):e157–66. 10.1016/j.jfo.2020.04.004 10.1016/j.jfo.2020.04.004 32381369

[70] Xu J, Zhao C, Kang Y. The formation and renewal of photoreceptor outer segments. Cells. 2024;13(16):1357. 10.3390/cells13161357 10.3390/cells13161357 PMC11352558 39195247

