# Supplementary material for: Optical coherence tomography in patients with major depressive disorder
Source: BMC Psychiatry. 2025 Apr 9;25:356. doi: 10.1186/s12888-025-06775-7 (PMC11983771; doi:10.1186/s12888-025-06775-7)
Supplement: Supplementary file 1 — Supplementary Material 1 [file 12888_2025_6775_MOESM1_ESM.docx]

**Supplementary material to**

**“Optical coherence tomography in patients with major depressive disorder”**

Table of content:

Page 1 Supplementary Table 1: Global peripapillary RNFL thickness from the circle scan around the optic nerve head.

Page 2-3 Supplementary Table 2: Total Volume and average thickness of the macular retinal layers within the 9 sector EDTRS grid (the fovea centered scan).

Page 4 Supplementary Figure 1: Subgroup analysis comparing (A) medicated and unmedicated patients with MDD to HC and (B) patients with a severe depressive episode without psychotic symptoms (F32.2) and a recurrent depressive disorder, with a current severe episode without psychotic symptoms (F33.2) to HC.

Page 5 Supplementary Figure 2: ROC curves for the present OCT data and the ERG parameters of our previous investigations in an overlapping MDD and HC sample.

**Supplementary Table 1:** Global peripapillary RNFL (pRNLF) thickness (in µm) from the GH grid for all three circle scans. Data of both eyes from all participants (31 MDD; 60 HC) are summarized as mean and standard deviation (SD) with additional bootstrapped 95% confidence intervals (CI). For eye comparisons (paired tests) 29 MDD and 56 HC provided data from both eyes (only *p*-values are shown). *P*-values were calculated by permutation tests on the differences of means. Significance level in brackets was not adjusted (#) for eye comparisons, for group comparisons an FDR procedure was applied. Relative differences in means are depicted in % (R vs. L and MDD vs. HC). Cliff’s delta (δ) was computed as non-parametric effect size estimation. Significant comparisons are emphasized by grey-shaded background.

Abbreviations: CI = confidence interval; FDR = False discovery rate; GH grid = Garway-Heath grid; HC = healthy controls; MDD = patients with major depressive disorder; L = left eye; NL = number of left eyes; NR = number of right eyes; N.S. = not significant; pRNFL = peripapillary retinal nerve fiber layer; R = right eye; SD = standard deviation; δ = Cliff's Delta effect size; * = significant.

| **Circle** | **Eye** | **MDD (N_R_=30, N_L_=30)** | | **HC (N_R_=59, N_L_=57)** | | **MDD  vs. HC** | **δ** | ***p*-value** |
| --- | --- | --- | --- | --- | --- | --- | --- | --- |
|  |  | **Mean (SD)** | **95% CI** | **Mean (SD)** | **95% CI** |  |  |  |
| 3.5 mm | R | 107.9 (7.99) | [104.83, 110.48] | 105.74 (8.51) | [103.66, 107.90] | +2.04% | 0.18 | 0.261 (N.S.) |
|  | L | 106.83 (8.33) | [103.93, 109.77] | 105.70 (8.07) | [103.72, 107.91] | +1.07% | 0.09 | 0.552 (N.S.) |
|  | R vs. L | +1.20% | *p*=0.004 (*)# | +0.32%; | *p*=0.458 (N.S.)# |  |  |  |
| 4.1 mm | R | 91.67 (7.03) | [89.04, 94.00] | 90.29 (6.85) | [88.63, 92.09] | +1.52% | 0.13 | 0.382 (N.S.) |
|  | L | 90.93 (6.99) | [88.40, 93.37] | 90.61 (7.33) | [88.77, 92.55] | +0.36% | 0.05 | 0.851 (N.S.) |
|  | R vs. L | +1.14% | *p*=0.022 (*)# | −0.06% | *p*=0.903 (N.S.)# |  |  |  |
| 4.7 mm | R | 79.93 (6.03) | [77.83, 82.10] | 78.86 (6.30) | [77.33, 80.51] | +1.36% | 0.11 | 0.438 (N.S.) |
|  | L | 79.63 (6.48) | [77.37, 81.93] | 78.92 (6.21) | [77.36, 80.61] | +0.90% | 0.10 | 0.619 (N.S.) |
|  | R vs. L | +0.78% | *p*=0.191 (N.S.)# | +0.14% | *p*=0.781 (N.S.)# |  |  |  |

**Supplementary Table 2:** Macular retinal layers from the ETDRS grid for both groups. The total volume (Vol. [mm³]) and the average thickness (Thick. [µm]; average thickness across 9 sector tiles) of the ETDRS grid is depicted in bold, alongside with the mean thickness data of the individual sector tiles. C0 corresponds to the fovea (1 mm diameter), sectors labeled with “1” corresponds to the para-foveal ring (3 mm diameter), sectors labeled with “2” correspond to the peri-foveal region (6 mm diameter). Peri- and para-foveal rings are divided into the superior (S), the nasal (N), the inferior (I) and the temporal (T) regions. Data are summarized as mean and standard deviation (SD) with additional bootstrapped 95% confidence intervals (CI). *P*-values for group comparisons were computed based on permutation tests for the differences in means. Significance level was adjusted according to an FDR procedure considering the number of layers. *P*-values < 0.05 are emphasized by grey-shaded background. Light grey-shading was used when significance was rejected after FDR procedure, dark grey-shading was used for significant comparisons with FDR adjustment included. Cliff’s delta (δ) was calculated as effect size. The relative deviation of the MDD mean from the HC mean is given in % (MDD vs. HC).

Abbreviations: CI = confidence interval; ETDRS grid = Early Treatment Diabetic Retinopathy Study; FDR = False discovery rate; GCL = ganglion cell layer; HC = healthy controls; I1 & I2 = para- and peri-foveal inferior sector; INL = inner nuclear layer; IPL = inner plexiform layer; MDD = patients with major depressive disorder; N = number of participants; N1 & N2 = para- and peri-foveal nasal sector; N.S. = not significant; ONL = outer nuclear layer; OPL = outer plexiform layer; PR+RPE = complex of the photoreceptor-retinal pigment epithelium; RNFL = retinal nerve fiber layer; S1 & S2 = para- and peri-foveal superior sector; SD = standard deviation; T1 & T2 = para- and peri-foveal temporal sector; Thick. = average thickness across 9 ETDRS sector tiles in µm; Vol. = total volume in mm³ of the ETDRS grid; δ = Cliff's Delta effect size; * = significant.

| **Layer/ measure** | | **MDD (N=31)** | | **HC (N=60)** | | **MDD vs HC** | **δ** | ***p*-value** |
| --- | --- | --- | --- | --- | --- | --- | --- | --- |
|  |  | **Mean (SD)** | **95% CI** | **Mean (SD)** | **95% CI** |  |  |  |
| **RETINA** | **Vol. [mm³]** | **8.72 (0.32)** | **[8.62, 8.84]** | **8.83 (0.36)** | **[8.75, 8.93]** | **−1.25%** | **−0.20** | **0.156 (N.S.)** |
|  | **Thick. [µm]** | **284.17 (10.61)** | **[280.56, 287.92]** | **288.29 (11.27)** | **[285.55, 291.14]** | **−1.43%** | **−0.22** | **0.089 (N.S.)** |
|  | C0 | 271.94 (20.14) | [264.62, 278.69] | 275.11 (18.96) | [270.55, 280.01] | −1.15% | −0.03 | 0.461 (N.S.) |
|  | S1 | 343.56 (13.80) | [338.97, 348.55] | 349.82 (14.27) | [346.27, 353.59] | −1.79% | −0.25 | 0.043 (N.S.) |
|  | S2 | 303.71 (10.91) | [299.90, 307.47] | 306.33 (14.50) | [302.76, 309.99] | −0.86% | −0.12 | 0.397 (N.S.) |
|  | N1 | 342.66 (14.58) | [337.69, 347.85] | 349.78 (15.42) | [345.73, 353.56] | −2.04% | −0.25 | 0.037 (N.S.) |
|  | N2 | 320.35 (12.70) | [316.11, 324.92] | 324.78 (15.15) | [321.12, 328.75] | −1.36% | −0.15 | 0.173 (N.S.) |
|  | I1 | 341.13 (14.15) | [336.42, 346.18] | 346.88 (13.67) | [343.54, 350.29] | −1.66% | −0.22 | 0.069 (N.S.) |
|  | I2 | 295.08 (13.19) | [290.87, 300.27] | 296.00 (12.52) | [292.98, 299.30] | −0.31% | −0.08 | 0.761 (N.S.) |
|  | T1 | 329.94 (13.57) | [325.31, 334.60] | 336.02 (14.02) | [332.51, 339.60] | −1.81% | −0.23 | 0.054 (N.S.) |
|  | T2 | 283.87 (11.61) | [280.16, 288.35] | 288.96 (13.30) | [285.61, 292.30] | −1.76% | −0.25 | 0.082 (N.S.) |
| **RNFL** | **Vol. [mm³]** | **0.91 (0.07)** | **[0.88, 0.93]** | **0.92 (0.08)** | **[0.90, 0.94]** | **−1.34%** | **−0.12** | **0.461 (N.S.)** |
|  | **Thick. [µm]** | **25.02 (1.60)** | **[24.49, 25.60]** | **25.32 (1.84)** | **[24.85, 25.78]** | **−1.20%** | **−0.11** | **0.453 (N.S.)** |
|  | C0 | 12.60 (2.19) | [11.74, 13.27] | 12.13 (1.93) | [11.64, 12.60] | +3.86% | 0.16 | 0.309 (N.S.) |
|  | S1 | 23.03 (2.02) | [22.34, 23.74] | 24.02 (2.29) | [23.43, 24.59] | −4.10% | −0.24 | 0.048 (N.S.) |
|  | S2 | 37.13 (3.45) | [35.97, 38.34] | 37.97 (4.06) | [36.99, 39.03] | −2.21% | −0.12 | 0.330 (N.S.) |
|  | N1 | 19.77 (1.66) | [19.27, 20.45] | 20.23 (1.79) | [19.80, 20.71] | −2.25% | −0.19 | 0.257 (N.S.) |
|  | N2 | 48.00 (5.08) | [46.27, 49.79] | 48.85 (5.35) | [47.49, 50.16] | −1.75% | −0.09 | 0.477 (N.S.) |
|  | I1 | 24.65 (1.72) | [24.10, 25.30] | 25.24 (2.45) | [24.62, 25.82] | −2.35% | −0.15 | 0.248 (N.S.) |
|  | I2 | 41.71 (5.96) | [40.08, 44.44] | 41.00 (5.35) | [39.70, 42.37] | +1.72% | 0.05 | 0.570 (N.S.) |
|  | T1 | 16.24 (0.66) | [15.98, 16.45] | 16.41 (1.06) | [16.14, 16.68] | −1.01% | −0.08 | 0.467 (N.S.) |
|  | T2 | 17.55 (0.92) | [17.24, 17.87] | 18.11 (1.13) | [17.83, 18.40] | −3.11% | −0.30 | 0.020 (N.S.) |
| **GCL** | **Vol. [mm³]** | **1.15 (0.08)** | **[1.12, 1.18]** | **1.15 (0.09)** | **[1.13, 1.17]** | **+0.43%** | **0.03** | **0.781 (N.S.)** |
|  | **Thick. [µm]** | **39.04 (2.79)** | **[38.09, 40.00]** | **38.90 (2.70)** | **[38.22, 39.59]** | **+0.35%** | **0.02** | **0.813 (N.S.)** |
|  | C0 | 15.66 (3.85) | [14.34, 17.00] | 15.20 (4.37) | [14.28, 16.52] | +3.06% | 0.13 | 0.610 (N.S.) |
|  | S1 | 55.23 (3.97) | [53.84, 56.58] | 55.06 (3.74) | [54.17, 56.05] | +0.30% | 0.03 | 0.856 (N.S.) |
|  | S2 | 37.40 (2.97) | [36.47, 38.56] | 36.56 (3.46) | [35.70, 37.42] | +2.30% | 0.12 | 0.273 (N.S.) |
|  | N1 | 52.68 (4.26) | [51.16, 54.13] | 53.17 (4.46) | [52.05, 54.25] | −0.94% | −0.08 | 0.627 (N.S.) |
|  | N2 | 41.19 (3.17) | [40.10, 42.29] | 40.95 (3.65) | [40.06, 41.92] | +0.58% | 0.05 | 0.769 (N.S.) |
|  | I1 | 55.11 (4.33) | [53.65, 56.58] | 54.97 (3.75) | [54.07, 55.93] | +0.26% | 0.02 | 0.858 (N.S.) |
|  | I2 | 35.60 (2.71) | [34.66, 36.53] | 35.16 (3.04) | [34.45, 35.97] | +1.24% | 0.11 | 0.514 (N.S.) |
|  | T1 | 50.42 (4.23) | [48.98, 51.85] | 50.59 (4.12) | [49.56, 51.65] | −0.33% | −0.03 | 0.862 (N.S.) |
|  | T2 | 37.56 (3.75) | [36.35, 38.98] | 38.06 (3.97) | [37.08, 39.08] | −1.31% | −0.10 | 0.583 (N.S.) |
| **IPL** | **Vol. [mm³]** | **0.94 (0.06)** | **[0.92, 0.96]** | **0.93 (0.06)** | **[0.92, 0.95]** | **+0.69%** | **0.05** | **0.636 (N.S.)** |
|  | **Thick. [µm]** | **32.66 (2.13)** | **[31.92, 33.40]** | **32.47 (1.96)** | **[31.99, 32.97]** | **+0.58%** | **0.05** | **0.652 (N.S.)** |
|  | C0 | 21.55 (3.71) | [20.27, 22.81] | 21.15 (3.73) | [20.28, 22.18] | +1.90% | 0.10 | 0.621 (N.S.) |
|  | S1 | 42.92 (3.07) | [41.84, 43.98] | 42.99 (2.77) | [42.29, 43.68] | −0.17% | −0.02 | 0.928 (N.S.) |
|  | S2 | 29.92 (2.20) | [29.16, 30.68] | 29.21 (2.65) | [28.53, 29.87] | +2.43% | 0.15 | 0.216 (N.S.) |
|  | N1 | 43.48 (3.18) | [42.34, 44.56] | 43.75 (3.00) | [43.00, 44.49] | −0.60% | −0.04 | 0.687 (N.S.) |
|  | N2 | 31.27 (2.33) | [30.45, 32.08] | 31.14 (2.69) | [30.50, 31.85] | +0.44% | 0.07 | 0.832 (N.S.) |
|  | I1 | 42.79 (2.98) | [41.73, 43.79] | 42.77 (2.67) | [42.12, 43.45] | +0.05% | 0.01 | 0.988 (N.S.) |
|  | I2 | 28.65 (2.09) | [27.92, 29.40] | 28.07 (2.22) | [27.51, 28.62] | +2.06% | 0.15 | 0.238 (N.S.) |
|  | T1 | 43.08 (3.68) | [41.81, 44.34] | 42.98 (2.77) | [42.30, 43.71] | +0.23% | 0.02 | 0.872 (N.S.) |
|  | T2 | 33.44 (2.73) | [32.53, 34.44] | 33.40 (2.58) | [32.76, 34.06] | +0.09% | −0.03 | 0.968 (N.S.) |
| **INL** | **Vol. [mm³]** | **0.98 (0.06)** | **[0.96, 1.00]** | **0.98 (0.06)** | **[0.97, 1.00]** | **−0.27%** | **−0.04** | **0.858 (N.S.)** |
|  | **Thick. [µm]** | **32.37 (2.19)** | **[31.65, 33.17]** | **32.46 (2.05)** | **[31.96, 32.99]** | **−0.29%** | **−0.05** | **0.836 (N.S.)** |
|  | C0 | 18.68 (4.53) | [17.15, 20.27] | 18.28 (4.66) | [17.19, 19.58] | +2.16% | 0.08 | 0.699 (N.S.) |
|  | S1 | 41.26 (4.09) | [39.94, 42.81] | 41.42 (2.95) | [40.67, 42.13] | −0.38% | −0.07 | 0.848 (N.S.) |
|  | S2 | 33.13 (2.60) | [32.32, 34.10] | 32.85 (2.08) | [32.33, 33.39] | +0.86% | 0.06 | 0.579 (N.S.) |
|  | N1 | 39.97 (3.67) | [38.84, 41.40] | 40.74 (3.71) | [39.84, 41.68] | −1.90% | −0.19 | 0.352 (N.S.) |
|  | N2 | 35.13 (2.18) | [34.35, 35.89] | 35.38 (2.48) | [34.79, 36.03] | −0.70% | −0.04 | 0.659 (N.S.) |
|  | I1 | 40.77 (3.71) | [39.63, 42.24] | 41.17 (3.51) | [40.33, 42.07] | −0.96% | −0.11 | 0.635 (N.S.) |
|  | I2 | 32.47 (2.56) | [31.60, 33.39] | 32.46 (2.23) | [31.93, 33.05] | +0.03% | −0.01 | 1.000 (N.S.) |
|  | T1 | 38.84 (3.32) | [37.73, 40.03] | 39.04 (3.07) | [38.23, 39.79] | −0.52% | −0.09 | 0.778 (N.S.) |
|  | T2 | 33.95 (2.42) | [33.13, 34.84] | 34.05 (2.31) | [33.48, 34.62] | −0.29% | −0.06 | 0.881 (N.S.) |
| **OPL** | **Vol. [mm³]** | **0.80 (0.07)** | **[0.77, 0.82]** | **0.78 (0.04)** | **[0.77, 0.79]** | **+2.15%** | **0.07** | **0.154 (N.S.)** |
|  | **Thick. [µm]** | **27.41 (2.58)** | **[26.61, 28.42]** | **26.60 (1.76)** | **[26.18, 27.06]** | **+3.03%** | **0.14** | **0.089 (N.S.)** |
|  | C0 | 27.15 (4.97) | [25.32, 28.74] | 24.70 (4.80) | [23.57, 25.96] | +9.92% | 0.30 | 0.023 (N.S.) |
|  | S1 | 35.11 (9.21) | [32.24, 38.83] | 33.05 (6.58) | [31.59, 34.97] | +6.26% | 0.05 | 0.229 (N.S.) |
|  | S2 | 26.95 (3.55) | [25.90, 28.39] | 26.14 (1.98) | [25.68, 26.67] | +3.11% | 0.02 | 0.181 (N.S.) |
|  | N1 | 30.56 (3.58) | [29.50, 32.03] | 30.94 (3.85) | [30.07, 31.99] | −1.21% | −0.06 | 0.693 (N.S.) |
|  | N2 | 27.50 (2.11) | [26.79, 28.24] | 27.43 (1.65) | [27.02, 27.85] | +0.26% | 0.00 | 0.881 (N.S.) |
|  | I1 | 31.58 (4.70) | [30.11, 33.42] | 31.06 (3.98) | [30.17, 32.19] | +1.67% | 0.04 | 0.577 (N.S.) |
|  | I2 | 26.23 (2.15) | [25.50, 27.00] | 25.78 (1.45) | [25.40, 26.13] | +1.72% | 0.08 | 0.263 (N.S.) |
|  | T1 | 32.26 (3.55) | [31.18, 33.65] | 31.02 (3.82) | [30.32, 32.47] | +4.00% | 0.23 | 0.157 (N.S.) |
|  | T2 | 27.24 (2.15) | [26.53, 28.00] | 26.65 (1.38) | [26.31, 27.00] | +2.24% | 0.15 | 0.124 (N.S.) |
| **ONL** | **Vol. [mm³]** | **1.74 (0.14)** | **[1.70, 1.79]** | **1.84 (0.19)** | **[1.79, 1.89]** | **−5.25%** | **−0.32** | **0.013 (N.S.)** |
|  | **Thick. [µm]** | **61.01 (4.69)** | **[59.47, 62.76]** | **64.77 (6.45)** | **[63.11, 66.42]** | **−5.82%** | **−0.38** | **0.003 (*)** |
|  | C0 | 89.82 (8.45) | [86.87, 92.84] | 96.20 (8.84) | [93.92, 98.38] | −6.63% | −0.42 | 0.002 (*) |
|  | S1 | 66.24 (9.74) | [62.34, 69.26] | 72.55 (10.25) | [69.79, 74.87] | −8.69% | −0.38 | 0.009 (N.S.) |
|  | S2 | 61.27 (5.91) | [59.21, 63.27] | 64.70 (7.39) | [62.82, 66.53] | −5.30% | −0.29 | 0.027 (N.S.) |
|  | N1 | 74.73 (6.12) | [72.67, 76.90] | 78.60 (9.75) | [76.10, 80.95] | −4.93% | −0.34 | 0.047 (N.S.) |
|  | N2 | 59.44 (5.16) | [57.68, 61.27] | 62.16 (7.50) | [60.31, 64.05] | −4.38% | −0.21 | 0.080 (N.S.) |
|  | I1 | 67.52 (8.53) | [64.37, 70.40] | 71.80 (8.63) | [69.65, 73.96] | −5.97% | −0.26 | 0.026 (N.S.) |
|  | I2 | 54.55 (5.24) | [52.77, 56.40] | 56.42 (5.87) | [55.00, 57.94] | −3.33% | −0.19 | 0.137 (N.S.) |
|  | T1 | 69.35 (6.13) | [67.15, 71.37] | 75.09 (8.36) | [72.92, 77.08] | −7.63% | −0.48 | <0.001 (*) |
|  | T2 | 57.65 (5.15) | [55.87, 59.47] | 60.95 (6.69) | [59.28, 62.67] | −5.42% | −0.29 | 0.017 (N.S.) |
| **ISOS+RPE** | **Vol. [mm³]** | **2.21 (0.05)** | **[2.19, 2.22]** | **2.24 (0.06)** | **[2.22, 2.25]** | **−1.25%** | **−0.26** | **0.033 (N.S.)** |
|  | **Thick. [µm]** | **72.48 (1.49)** | **[71.96, 72.99]** | **73.40 (2.00)** | **[72.91, 73.93]** | **−1.25%** | **−0.27** | **0.023 (N.S.)** |
|  | C0 | 86.89 (2.76) | [85.97, 87.89] | 88.08 (2.97) | [87.33, 88.83] | −1.36% | −0.23 | 0.070 (N.S.) |
|  | S1 | 79.74 (1.84) | [79.05, 80.31] | 80.81 (2.42) | [80.21, 81.44] | −1.32% | −0.26 | 0.034 (N.S.) |
|  | S2 | 78.13 (1.71) | [77.41, 78.61] | 78.99 (2.29) | [78.42, 79.56] | −1.09% | −0.21 | 0.078 (N.S.) |
|  | N1 | 81.35 (2.09) | [80.68, 82.13] | 82.35 (2.53) | [81.70, 82.97] | −1.21% | −0.24 | 0.066 (N.S.) |
|  | N2 | 77.94 (1.77) | [77.23, 78.48] | 78.92 (2.56) | [78.25, 79.53] | −1.24% | −0.25 | 0.069 (N.S.) |
|  | I1 | 78.81 (2.02) | [78.07, 79.49] | 79.83 (2.44) | [79.21, 80.43] | −1.28% | −0.24 | 0.053 (N.S.) |
|  | I2 | 76.03 (2.04) | [75.26, 76.69] | 77.11 (2.48) | [76.47, 77.72] | −1.40% | −0.24 | 0.044 (N.S.) |
|  | T1 | 79.81 (1.96) | [79.10, 80.47] | 80.92 (2.24) | [80.36, 81.48] | −1.38% | −0.27 | 0.023 (N.S.) |
|  | T2 | 76.63 (1.75) | [75.94, 77.16] | 77.75 (2.27) | [77.17, 78.30] | −1.44% | −0.29 | 0.019 (N.S.) |


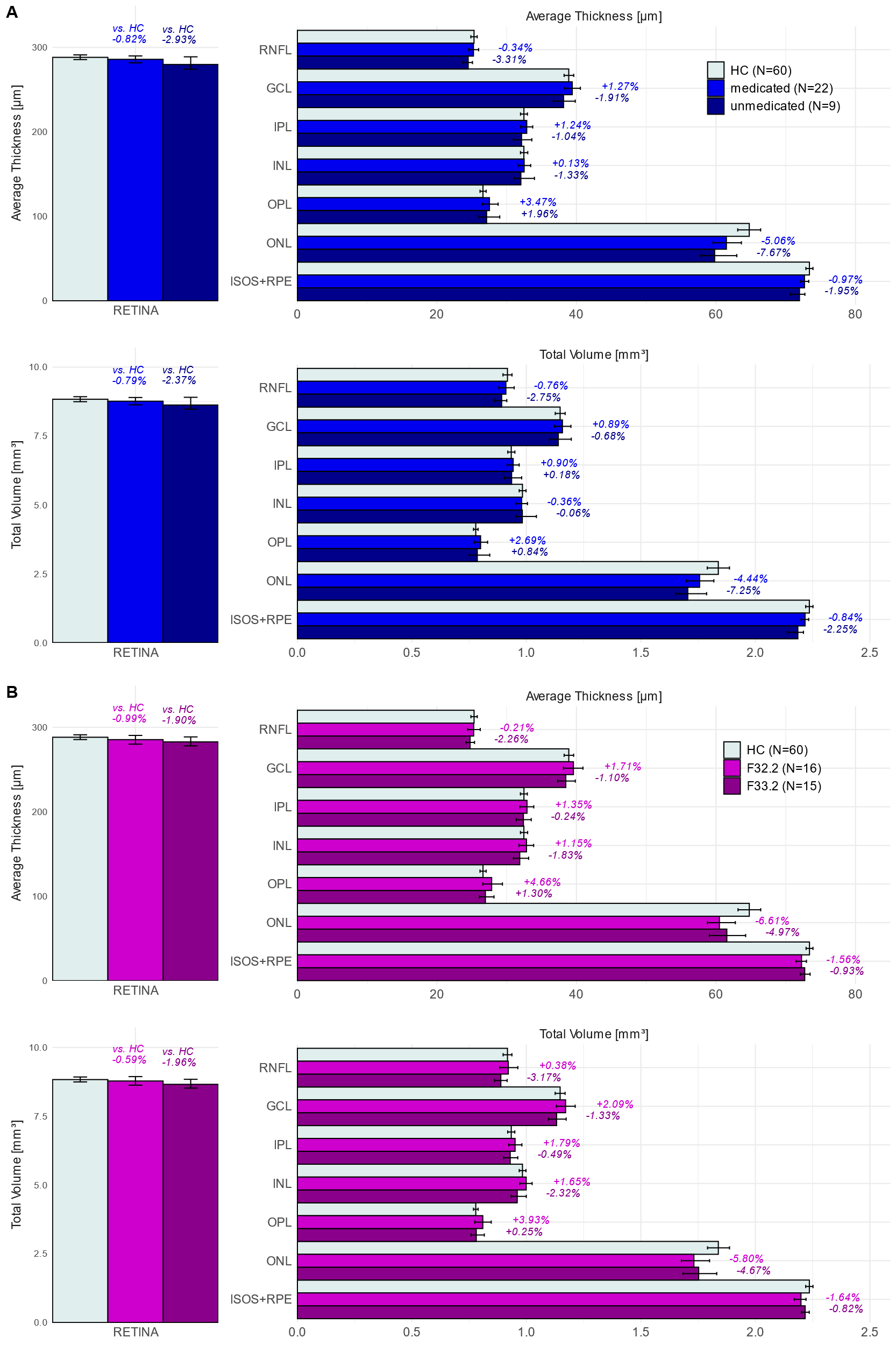


**Supplementary Figure 1:** Subgroup analysis comparing (A) medicated (N=22) and unmedicated (N=9) patients with MDD to HC and (B) patients with a severe depressive episode (F32.2; N=16) and patients with a recurrent depressive episode (F33.2; N=15) to HC. The mean and 95% CI (error bars) of the average thickness and total volumes of all retinal layers from the ETDRS grid are depicted for both groups. Relative deviations of the MDD’s from HC’s mean in % are annotated.

Abbreviations: CI = confidence interval; ETDRS grid = Early Treatment Diabetic Retinopathy Study; GCL = ganglion cell layer; HC = healthy controls; INL = inner nuclear layer; IPL = inner plexiform layer; MDD = patients with major depressive disorder; N = number of participants; ONL = outer nuclear layer; OPL = outer plexiform layer; ISOS+RPE = complex of the inner and outer segments of the photoreceptors and the retinal pigment epithelium; RNFL = retinal nerve fiber layer.


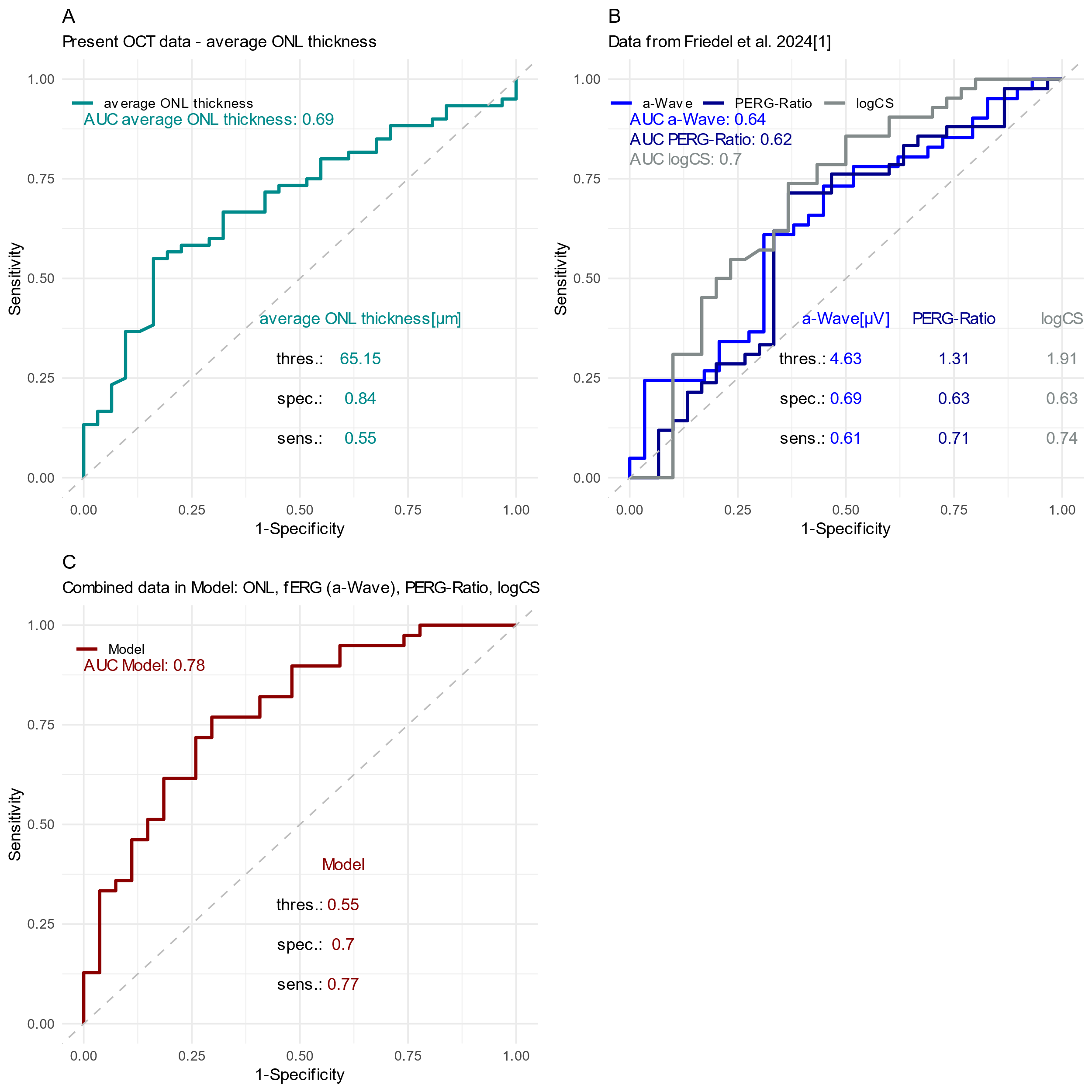


**Supplementary Figure 2:**

Receiver operating characteristic (ROC) curves for the present OCT data (average ONL thickness) (**A**), the electrophysiological measures (PERG ratio and fERG a-wave), and the psychophysiological data (contrast sensitivity in logCS) from our previous study (Friedel et al., 2024[1]) in an overlapping sample (N_MDD_ = 27, N_HC_ = 39) (**B**), as well as for a generalized linear model incorporating all parameters (**C**) are shown. The area under the curve (AUC), the best performing cut points (threshold values = thres.) and their corresponding sensitivity (sens.) and specificity (spec.) values are annotated.

Abbreviations: AUC = area under the curve; logCS = contrast sensitivity in logCS; OCT = optical coherence tomography; ONL = outer nuclear layer; PERG-Ratio = pattern electroretinogram ratio; thres = threshold value; sens = sensitivity; spec = specificity.

References

[1] Friedel EBN et al., “Reduced Contrast Sensitivity, Pattern Electroretinogram Ratio, and Diminished a-Wave Amplitude in Patients with Major Depressive Disorder,” European Archives of Psychiatry and Clinical Neuroscience, May 28, 2024, https://doi.org/10.1007/s00406-024-01826-8.
